# Key Factors to Improve the Outcome of Retinal Reattachment Surgery in Proliferative Vitreoretinopathy and Proliferative Diabetic Retinopathy

**DOI:** 10.1155/2017/2323897

**Published:** 2017-07-09

**Authors:** Svenja Deuchler, Hanns Ackermann, Pankaj Singh, Thomas Kohnen, Clemens Wagner, Frank Koch

**Affiliations:** ^1^Vitreoretinal Unit, Department of Ophthalmology, Goethe University, Frankfurt am Main, Germany; ^2^Institute of Biostatistics and Mathematical Modelling, University Hospital, 60590 Frankfurt am Main, Hessen, Germany; ^3^Department of Ophthalmology, Goethe University, Frankfurt am Main, Germany; ^4^VRmagic GmbH, 68167 Mannheim, Baden-Württemberg, Germany

## Abstract

**Introduction:**

For management of complicated retinal detachments, a pars plana vitrectomy with temporary silicone oil (SO) fill is the method of choice. According to literature, the retinal redetachment rate varies between <10% and >70% with around 36% in our own group (retrospective data analysis, *n* = 119 eyes).

**Methods:**

The main goal was to reduce the retinal redetachment rate. Standard operating procedures (SOPs) and evaluation protocols (EVALPs) were developed to prospectively analyse risk factors. Lab analysis of SO was performed, and the role of surgical experience was evaluated and investigated with Eyesi®.

**Results:**

We achieved a significant reduction of the retinal redetachment rate (to 6.80%, *n* = 101, *p* = 0.002). After surgery with SO injection, neither further membrane peeling (in 16.5%) nor retinal laser coagulation (in 100%) during revision surgery had a significant effect on the reattachment rate (*p* = 0.167, *p* = 0.23), while extensive additional laser coagulation reduced visual acuity (*p* = 0.01). A 3-port approach had to be set up to complete SO removal. A difference in success rate depending on surgical experience was confirmed, and the performance in Eyesi correlated with that in the patients' eye.

**Conclusions:**

A SOP- and EVALP-based management and new strategies to secure the surgical performance seem to be essential for successful surgery.

## 1. Introduction

The most common complication of retinal reattachment surgery with a preliminary silicone oil fill is a retinal redetachment after the removal of the silicone oil. In literature, the redetachment rates vary between less than 10% and over 70%.

The cause analysis is “poor” and not sufficient to draw clear-cut conclusions to improve the success rate [1–13]. This poses the question, which parameters should be sought differently and improved upon in order to systematically reduce the rate of redetachments of the retina after the removal of silicone oil and to also check to what extent this is possible.

Goezinne et al. [11] describe the occurrence of a retinal redetachment within the first 3 months after oil removal in two-thirds of all eyes, and only 10% of the redetachments occurred later than 6 months after oil removal.

The immediate assessment of the actual condition of the retina and its environment after primary reattachment service is obviously critical and difficult at the same time. It is otherwise unexplainable, why redetachments after silicone oil removal occur very early if not immediately after [14]. As described by others [15, 16], our own retrospective data showed that in those eyes where redetachments occurred, this complication was not at all expected. Moreover, in two-thirds of the affected eyes, this occurred within the first four weeks after oil removal.

Decisive for the follow-up and the success rate of retinal detachment surgery are concomitant circumstances. Traditionally, these flow into the so called ^17^proliferative vitreoretinopathy [PVR] classification [17–19] and a variety of surgical consequences from this classification. Since in a proliferative diabetic retinopathy (PDR), there are many similarities with the PVR process; Kroll et al. introduced “fPDVR” in 2007 [19] which will be used in this work as P(D)VR from now.

As more damaged retinal tissue has to be removed (retinectomized) to reattach healthier retinal tissue, the risk will be higher that residual retina will also start to shrink and cannot be preserved permanently.

Performing a 360° laser coagulation before draining the silicone oil increases the safety of a permanent retina attachment [20]. The adequately performed laser coagulation reduces the redetachment rate after the removal of oil from 58% to 26%.

In De Silva's work, the probability of poor vision < 20/400 (corresponding to <1/50, threshold of legal blindness) increases with the increase of severity in the P(D)VR classification (by 15% in each of the classification degrees: stages A, B, C, etc.).

In Unlue et al.'s work [14], redetachment risk increases with the increase of severity in the P(D)VR classification; 9.5% with P(D)VR C, 25% with P(D)VR D, over 33% for tractional retinal detachment/trauma, and >37% with giant retinal tears.

The most recent studies of general risk factors fundamentally relevant for the successful establishment of a primary rhegmatogenic ablation retinae include the work published by Jiang et al. [21, 22], Kon et al. [23], and Rodríguez de la Rúa et al. [24]. In a retrospective multicenter study by the European Vitreoretinal Society (EVRS), published under the lead of Adelman et al. [25], substantial general risk factors were concluded from over 7500 eyes: choroidal detachment, significant hypotony, P(D)VR stage C-1, distribution of detached retina over all 4 quadrants, and the size/type of retinal holes (large/giant tears). In the EVRS study in which not only pars plana vitrectomy (PPV) but also buckling surgery was considered, the condition “retinal detachment before or after cataract surgery” was of secondary importance.

In Pavlovic et al.'s work [26], it is quite obvious how essential the assessment of a “clinically stable retinal reattachment” is for the decision whether to remove silicone oil or not (at a certain time) and also for the expected result. Depending on the more or less correct detection of the actual condition of all tissues, a redetachment occurs in 8% to 53%. Pavlovic et al. also stress the importance of laser coagulation.

Gupta and coworkers [27] have followed the progress of treatment options over 10 years in 346 eyes and found that when removing bloody vitreous or vitreous-causing retinal traction, both the anatomical success rate and visual acuity have increased steadily [28–30], most likely because vitreoretinal service was offered promptly (no later than 6 weeks). As many others in literature, they point out the need to meticously remove blood and diseased vitreous in the outermost periphery, particularly when dealing with proliferative tissue activity.

**Figure 1:**
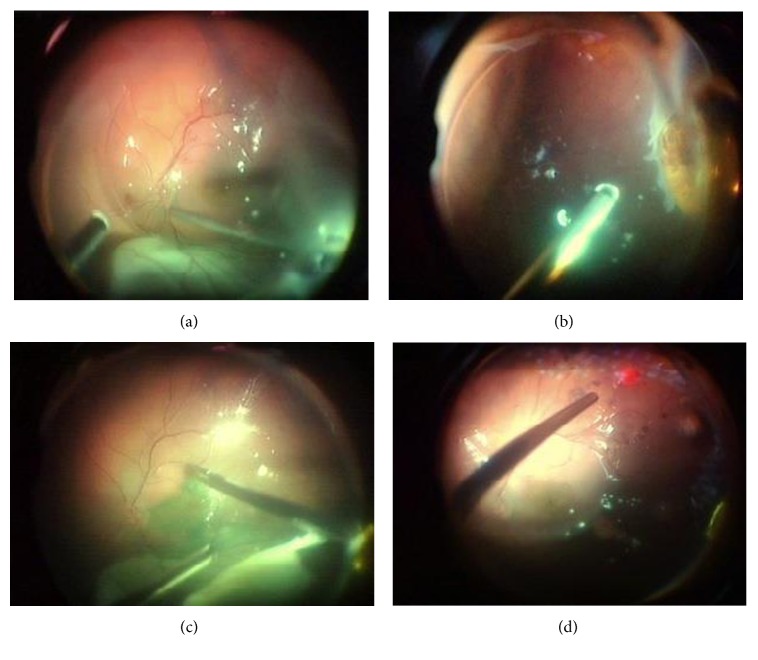
Scenes to document essential surgical steps: (a) filling of perfluorocarbon liquids (PFCL); (b) trimming necrotic retina tissue edges; (c) peeling of the inner limiting membrane after staining with indocyanine green; (d) laser coagulation of the retina edges after exchange of the PFCL against air before instillation of silicone oil.

Later, we will discuss that the shift from 20 g to smaller gauges, down to 27 gauge, includes an intrinsic risk-performing surgery less complete during primary service (Figure 1) which implicates different considerations for the revision surgery with intended silicone oil removal.

Since the comparability of known strategies taken from literature about the care and management of complicated retinal detachment as well as a variety of associated parameters was very unsatisfactory, and the results or success and failure rates of existing data were difficult to measure, the aim of this study is to improve the results using standard operation procedures (SOPs) with detailed evaluation protocols (EVALPs) and to show essential steps to achieve this goal.

## 2. Material and Methods

In the Department of Ophthalmology at the Goethe University in Frankfurt am Main, 119 eyes suffering from a complicated retinal detachment had been serviced with preliminary silicone oil injection at the end of a PPV. Data were retrospectively tracked and stored in an Excel database (Microsoft® Excel® for Windows, 2010; Excel for Mac, 2011). Surgeries performed by 12 vitreoretinal surgeons were included.

Finally, 101 eyes with sufficiently long follow-up documentation were evaluated and compared with patients prospectively followed up from there. In the majority of these retrospective cases (74 of 101), the silicone oil was drained via 2 channels (passive drainage) once fundoscopy had shown complete reattachment of the retina under the oil fill.

**Figure 2:**
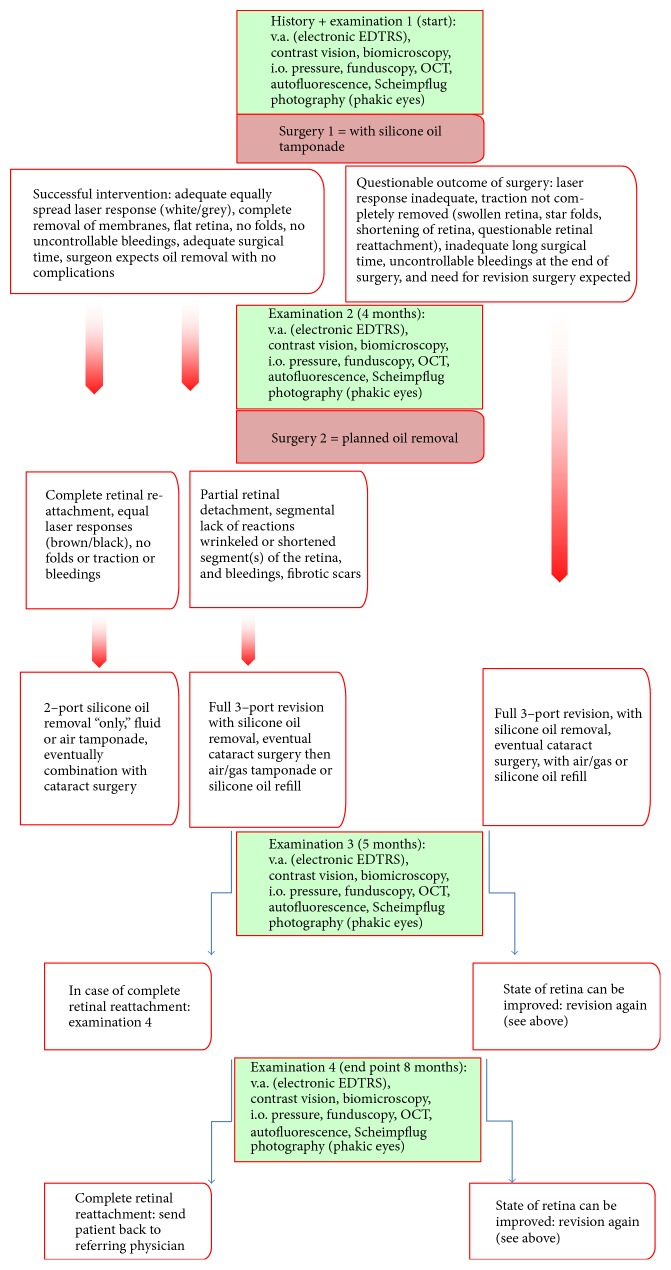
Standard operation procedure for the surgery of complex retinal detachments with preliminary silicone oil tamponade (initial version). EDTRS = international standard to present visual acuity (v.a.) values; biomicroscopy = examination of the anterior segment of the eye, for example, the lens; i.o. pressure = measurement of the pressure in the eye; funduscopy = examination of the retina; OCT: optical coherence tomography = noninvasive “cutting through” the retina using light wavelengths; autofluorescence; special photography of the retina RPE complex; Scheimpflug photography; special measurement of lens changes, related, for example, to aging, diabetes mellitus, inflammation, and trauma.

In about 36% of the silicone oil-filled eyes of our patients, a variety of complications after the removal of the oil led to one or more further surgical procedures. There were various reasons found to explain the origin of these complications. We tried to rank their importance: first of all, the quality of documentation of the surgical procedure [31]. Other reasons included the surgeon's choice of primary and secondary care, the surgical experience (years of experience in the vitreoretinal service), the condition (thickness) of the retina, attachment/detachment of the macula/fovea, the number and quality of laser coagulation, the stage of P(D)VR activity, and the quality and completeness of peeling (epiretinal membranes/ILM). After identifying the causes for the development of recurring retinal complications and retinal detachment, a proposal for a standard surgical procedure (SOP) was developed (Figure 2).

It was based on the protocol of a prospective study (*n* = 103 eyes) with an open, controlled study design and the main goal to determine the failure rate. The SOP was revised during the prospective continuation of the study, considering aspects known from literature and those from our own retrospectively collected data.

The study was approved by the ethical review committee, Goethe University in Frankfurt am Main (IRB decision number E 190/11, transaction number 403/11). This study was conducted in accordance with the tenets of the Declaration of Helsinki. Patients' records were pseudonymized and deidentified prior to statistical analysis.

All the analyses were performed using BiAS V10.12 [32] for Windows, IBM SPSS Statistics V22 and the R package V3.1–120 [33].

The data were analysed using numerous statistical tests [32–44]. The case count calculation was performed on the basis of the binomial test. A double-sided binomial test (Fisher's F distribution with rejection range *p*=2^∗^ (p/2)) was used to test the null hypothesis H0 (theta0 = 0.3564) against the alternative using theta0-thetaA ≤ 0.1782 and a 95% binomial confidence interval for the thetaA fraction. The recheck of the null hypothesis was repeated for the operator subgroups. The χ^2^-contingency panel test was performed in the presence of categories. The age distribution of the patients was compared using the 2-sample *t*-test in the modified form of the Welch test (for variance inequality). The necessary normal distribution of the values was proven by the Kolmogorov and Smirnov tests. The *F*-test for the comparison of two variances in two-sided questions was used to check the performance fluctuations between the most experienced and the inexperienced surgeon with and without virtual reality training in the Eyesi. In order to investigate which influence criteria were most likely to be responsible for the statistically significant reduction of the retinal detachment rate, a comparison was made by means of multiple regression.

Whether there was a statistically significant difference in the best postoperative visual acuity was tested using the Wilcoxon-Mann–Whitney *U* test. The predictors for the postoperative visual acuity (=dependent variable) were defined according to the above mentioned multiple regression method.

A correlation calculation according to Spearman was carried out to check the correlation between the risk factors which were derived from the retrospective analysis. The evaluation of the correlation coefficient (rho) was made using the effect strength according to Evans as follows: <0.2: poor; 0.2–0.4: weak; −0.4–0.6: moderate; 0.6–0.8: strong; and >0.8: optimal. To check the statistical significance (*p* < 0.05) of the results, a two-page test with Edgeworth approximation was obtained.

Our retrospective study data with *n* = 119 patients showed a retinal redetachment (failure) rate after removing the silicone oil from the vitreous cavity of 34%. The main goal was to reduce the failure rate by at least 50%.

This resulted in the minimum relevant difference of delta0 = −0.17. With the error probabilities of *α* = 0.05 and *β* = 0.1 and a power of 90%, a sample size of at least *n* = 69 patients was needed for a 2-sided question.

The following influencing variables were included in the EVALPs of the prospective study and evaluated according to their significance in the course of the observations:

- Analysis of visual acuity: visual acuity describes the perception of patterns and contours and is primarily tested for distance (5 m measuring distance) and proximity (30 cm), so that the person concerned can verifiably recognize letters or signs
- Quality of vision: for better measurement of surgical results, we initiated the systematic use of the “Central Vision Analyser (CVA).” The detection of visual quality with the Vimetrics® Central Vision Analyser (CVA) under mesopic and photopic conditions
- Influence of eye length: influence of the deviation from a normal eye length (emmetropia) service documentation: detailed data were collected in an evaluation protocol (EVALP)
- Membrane peeling: staining of membranes and extent of PVD, ERM, and ILM removal
- Laser: distribution and intensity
- Structure of the retina/P(D)VR: grading of the retina state according to the classification of the Retina Society
- Total operational supply in one hand: number of surgeons providing service for primary reattachment surgery and revision surgery with silicone oil removal
- Influence of the extent of practical experience (years of experience in the vitreoretinal service): vitreoretinal experience in years and systematically augmented skills (e.g., in Eyesi) are independent important criteria for the success rate
- Degree of retinal detachment: involvement of the macula
- Retinal holes: number, size, and condition of retina holes (e.g., enrolled edges and stiffness)
- Retinotomy and retinectomy: removal of retinal tissue; location and extent
- Influence of simulator training on the surgical performance: relation of surgical performance between surgery in the Eyesi surgical simulator and in the patients' eye and role of warming up in the simulator before going to the OR. By randomization, the surgeon either went immediately to the operating room or warmed-up by going through a short simulator training beforehand. Evaluation by two independent observers was performed to guarantee intragrader and intergrader consistency.

## 3. Results

Both in the retrospectively and prospectively compiled datasets, only the eyes with permanently attached retina after silicone oil removal were considered to be a success. Retrospectively, from 119 patients, 18 patients had to be excluded for the final calculation of the statistical analysis due to inadequate follow-up. 101 patients (*n* = 101) had an adequate observation period of 4 months after the oil drain and could be included in the analysis. There was a failure in *n* = 36 patients, consisting of retinal redetachment (*n* = 32) or a need for permanent oil fill (*n* = 4) of the eye. Overall, the failure rate was 35.64%.

From this, the null hypothesis H0 (theta = 0.3564) was deduced. H0 should be rejected with the aim of reducing the failure rate by at least 50% to 17.82% or less. This resulted in the minimum relevant difference of delta0 = −0.1782.

After evaluating the prospective data, a study inclusion of *n* = 113 patients was possible. *n* = 10 patients did not complete the study (dropouts). Thus, *n* = 103 patients were prospectively evaluated. In this case, a failure rate of *n* = 7, consisting of the number of redetachments of the retina (*n* = 3) and the number of continuous oil tamponades (*n* = 4), was evaluated.

With the aim of achieving a failure rate of <17.82% and the minimal relevant difference of delta0 = −0.1782, the null hypothesis could be rejected with *p* = 0.002 using the double-sided binomial test [36, 41]. The failure rate was reduced to 6.80%.

### 3.1. Calculation of the Confidence Interval

After rejecting the null hypothesis with *p* = 0.002, a binomial confidence interval for theta (0.07) with *p* = 0.95 was calculated.

This was valid for the proportion theta in *n* = 103 study participants and a failure rate of *k* = 7 patients from 0.027759 to 0.135022. That is, 95% of the failure rate was found to be between a lower limit of 2.77% and an upper limit of 13.50%, and thus a maximum failure rate of 13.50% according to the prospective method SOPs can be expected [41].

### 3.2. Comparison of Retrospective versus Prospective Study Populations

Prior to evaluating possible impact criteria on the failure rate, the two study groups were tested for homogeneity (Table 1).

A χ^2^ contingency panel test [35, 41] was carried out in the analyses concerning the demographics and characteristics of the participants in the course of classes (exception of age distribution).

### 3.2.1. Age

In order to determine whether the two groups (retrospective versus prospective) were based on the same age distribution, the 2-sample *t*-test was performed.

First, prerequisites for the 2-sample *t*-test were checked. The Kolmogorov-Smirnov test was carried out to test for the normality of distribution. The zero hypothesis was “The age distribution corresponds to a Gaussian distribution.” This could be maintained for both groups with *p* > 0.10. As a further assumption for the 2-sample *t*-test, homogeneity of the variances was checked using the *F*-test, which falls below the threshold of *α* = 0.10 with *p* = 0.0005 here, so that equality of the variances cannot be assumed. Thus, the 2-sample *t*-test was applied using the modified form of the Welch test: the *p* value is 0.0005, so that it could be assumed from different age distributions [41, 45].

### 3.2.2. Gender

Of the 101 fully evaluable patient eyes of the retrospective study section, 65 (64.36%) eyes were of men and 36 (35.64%) of women. 103 eyes were prospectively evaluated: 66 (64.08%) of men, 37 (35.92%) of women.

This showed no statistically significant difference with *p* = 0.99. Consequently, there was a comparable gender distribution with approximately twice as many male than female patients in both groups.

### 3.2.3. Right/Left Eyes

A similar distribution of the affected eye was also present: retrospectively, 50 (49.5%) right eyes and 51 (50.5%) left eyes were affected by the retinal detachment, prospectively 57 (55.34%) right eyes and 46 (44.66%) left eye (*p* = 0.49).

### 3.2.4. Fovea Situation

As more of the macula is affected by the retinal detachment, the more questionable the recovery of visual acuity and vision is. Therefore, macular involvement is a critical parameter. OCT technology can assist the assessment.

Retrospectively, there was no adequate data on the condition of the fovea preoperatively in 5 eyes (4.95%). In some cases, the statement was not sufficiently documented; ultimately, there was a detachment of the posterior pole in 60 eyes (59.41%), partial detachment of the fovea in 15 eyes (14.85%), and in 21 eyes (20.79%), the fovea was attached preoperatively.

In the prospective series, all 103 eyes showed definite information on the condition of the fovea: in 63 (61.16%) of the eyes, fovea was detached, in 18 eyes (17.48%), the fovea was underwashed by fluid (washed out), and in 22 eyes (21.36%), the fovea was preoperatively attached.

There was no significant difference between the two studies (*p* = 0.94). In both groups, the fovea was affected from the retinal detachment in four-fifths of all cases.

### 3.2.5. Retinal Redetachment Rate

In the retrospectively followed group (permanent retinal reattachment in 65 patients = 64.36%), an early retinal redetachment after primary attachment developed within 1 month after the oil drainage in 21 eyes (20.79%), redetachment occurred in 4 eyes (3.96%) after 4 months (3.96%), and later in 7 eyes (6.93%), 4 eyes (3.96%) needed a permanent oil tamponade to stabilize the retina.

After the development and use of the SOP and the evaluation curves in the prospectively followed group, the retina of 96 of 103 eyes (93.21%) stayed permanently attached over the entire period of follow-up. In each group, within 1 month, after 1 to 4 months, and after 4 months, one eye (0.97%) developed a redetachment; a permanent oil fill was needed in 4 eyes (3.88%).

The difference between the retrospective and the prospective study with *p* = 4 × 10^−6^ was highly significant.

### 3.2.6. Surgeons' Skill

In the retrospective group, 16 (15.84%) interventions were performed by the most experienced colleagues (experience > 25 years), in 36 eyes (35.65%) by surgeons with experience of >7 and in 49 eyes (48.51) from surgeons with 1 to 7 years experience.

In the prospective group, 24 eyes (23.30%) were performed by the most experienced surgeons, 54 (52.43%) by surgeons with >7 years of experience and 25 eyes (24.27%) by surgeons with 1 to 7 years of experience.

Between the two studies, there was a significant difference in *p* = 0.002 with regard to operational experience classes. For this reason, we retested the primary parameter “reduction of the failure rate by 50%” for the 3 subgroups with different surgical experience, in order to break down the effect of the experience profile on the main target value “permanently attached retina” (Table 2).

### 3.2.7. Timing of Cataract Surgery

Retrospectively, 29 eyes (28.71%) were operated after a previous cataract surgery (pseudophakic retinal detachment). In 8 eyes (7.92%), a combined phacoemulsification with implantation of a posterior chamber lens and a 3-port PPV was performed for the retinal reattachment maneuver using a preliminary silicone oil tamponade. In 35 eyes (34.65%), the lens was replaced between initial and revision surgery or during the revision surgery when silicone oil was removed and cataract surgery was performed at a later time in 29 eyes (28.72%). Prospectively, the rate of pseudophakic retinal detachment was 39.8% (41 eyes); in 3 cases, the PPV was combined with cataract surgery, another 41 eyes (39.8%) received a lens exchange together with the removal of silicone oil, and in 18 eyes (17.48%), it was performed later. There was no significant difference between these two groups (*p* = 0.06).

### 3.2.8. P(D)VR Classifications/Staging

The classification of proliferative (diabetic) vitreoretinopathy (P(D)VR) stages is based on the classification of the Retina Society 1983 and the Kroll classification of 2007 [17–19, 46, 47].

There were no retrospective reports in 10% of the eyes (10 eyes, 9.9%). In the PVR stage D2/D3/PDR stage D1 were 11 eyes (10.89%), in the PVR stage D1/PDR-C3 stage 9 eyes (8.91%), in the PVR-C3 and PDR-C3 (14.8%), in PVR-C2/PDR-C1 stage 18 eyes (17.82%), in PVR-C1/PDR-B stage 12 eyes (11.88%), and in PVR-AB/PDR-A stage had 26 eyes (25.75%).

All eyes were prospectively evaluated with 7 eyes (6.8%) in the most advanced stage PVR D2/D3/PDR D1, 14 eyes (13.59%) in PVR stage D1/PDR-C3 stage, 20 eyes (12.72%) in the PVR-C1/PDR-C2 stage, 16 eyes (15.53%) in the PVR-C2/PDR-C1 stage, and 33 eyes (32.04%) in the PVR-AB/PDR-A stage.

There was no significant difference between the two groups when staging the P(D)VR (*p* = 0.71).

### 3.2.9. Viscosity of the Silicone oil

Retrospectively, 80 eyes (79.21%) were filled with 5000 mPa·s of silicone oil, 4 eyes (3.96%) with 4300 mPa·s of silicone oil, and 13 eyes (12.87%) with 2000 mPa·s of silicone oil. In 4 eyes (3.96), no data were found on the type of oil used. Prospectively, 5000 mPa·s of oil were used in 69 eyes (66.99%), 4300 mPa·s in 6 eyes (5.83%), and 2000 mPa·s in 28 (27.2%) eyes. Based on the different manufacturing and marketing of silicone oils, the choice of oils differed significantly (*p* = 0.01).

### 3.2.10. Laser Coagulation

The intra- and postoperative assessment of the quality of the laser coagulation affects the planning of any further procedure.

Does laser coagulation has to be performed after primary retinal detachment service and before revision surgery with oil removal or during revision surgery, directly before or after the removal of oil, and what affects the anatomical outcome and how does visual function respond to it? A thickened (swollen) retina may not be lasered at all during surgery, a thinned retina is also associated with the risk of not being adequately linked to the pigment epithelium, or even being inadvertently penetrated by the laser (iatrogenic laser holes).

The evaluation of the laser coagulation was based on the reasonable number of laser spots as well as their quality (adequate responding). Retrospectively, lasers were classified as very good = grade “6” for 10 eyes (9.9%), grade “5” for 27 eyes (26.73%), grade “4” for 20 eyes (19.8%), grade “3” for 21 eyes (20.79%), grade “2” for 11 eyes (10.89%), and grade “1” for 7 (6.94%) eyes. In 5 eyes (4.95%), no adequate data were found.

Prospectively, grade “6” for 6 eyes (5.82%), grade “5” for 30 eyes (29.13%), grade “4” for 33 (32.04%), grade 3 for 24 (23.3%), grade “2” for 9 eyes (8.74%), and grade “1” for one eye (0.97%). There was no significant difference (*p* = 0.11), and the distribution was approximately the same in both groups.

### 3.2.11. Membrane Peeling during Primary Surgery

P(D)VR often affects the interface in the macula as well as outside of the macula. During primary service as well as revision surgery, both the removal of macula pucker tissue and all PVR membranes responsible for tissue contraction (e.g. star folds) have to be carefully and repeatedly evaluated.

The extent of membrane peeling performed during the primary retinal reattachment surgery was also classified into categories:

- Category 4 = VB + PVD + ERM + ILM peeling
- Category 3 = VB + PVD + ERM peeling
- Category 2 = VB + PVD
- Category 1 = VB.

The measures or points of value are additive.

Retrospective category 1 = vitreous body (VB) removal in 3 eyes (2.97%), category 2 = VB removal plus generation of a posterior vitreous detachment (PVD) in 72 eyes (71.29%), category 3 = in addition to category 2 = peeling of epiretinal membranes (ERM) in 22 eyes (21.78%), and category 4 = in addition to category 3 peeling of the membrana limitans interna (ILM) performed in 4 eyes (3.96%).

Prospective category = comprehensive peeling of category 4 was carried out in 79 eyes (76.69%), while all structures except the ILM were peeled (category 3) in 11 eyes (10.67%) and (category 2) in 12 eyes (11.65%); a VB removal and a PVD were performed, and (category 1) in 1 eye (0.97%), only the removal of vitreous body was mentioned.

In the prospective group, the surgeons performed significantly more membrane peelings (*p* < 10^−6^).

### 3.2.12. Consistency of the Surgeon

Retrospectively, initial and follow-up in 26 interventions were carried out by one surgeon (25.74%), 2 different surgeons were involved in 19 eyes (18.81%), and 3 or more surgeons in 55 eyes (54.46%). For one intervention (0.99%), there was no useable data.

Prospectively, in 30 eyes (29.13%), surgeries were in one surgeon's hand; in 16 eyes (15.53%), in the hands of 2 different surgeons; and the remaining surgeries were accomplished by 3 or more surgeons in 57 eyes (55.34%). There was no significant difference (*p* = 0.76).

### 3.2.13. Revision Type: 2-Port/3-Port

The qualitative changes of the oils (manufacturing/marketing reasons) had significant impact on the prospective protocol of the study, while retrospectively, it was freely chosen between the two approaches (103 eyes), with a 2-port approach in 73.27% (74 eyes) and with a 3-port approach in 21.78% (23 eyes, no data in 5 eyes = 4.95%). Prospectively, a 3-port approach had to be set up for all 103 (100%) eyes because of the necessity for multiple rinsing of the vitreous cavity to guarantee as complete as possible removal of the oil (*p* = 1 × 10^−6^).

Once in the eye (3-port approach), additional laser and/or peeling could be considered.

### 3.2.14. Membrane Peeling in the Revision Surgery

Retrospectively, 18 eyes (17.82%) were peeled in the revision surgery with silicone oil removal from the vitreous cavity, and no membrane peeling was performed in 81 eyes (80.20%). For 2 eyes (1.98%), there was no useable data.

Prospectively, 17 eyes were peeled (16.51%), 83 eyes (80.58%) were not peeled in the revision, and there was no useable data for 3 eyes (2.91%).

There is no statistically significant difference (*p* = 0.82).

### 3.2.15. Myopia

An increased eye length (high myopia) is often accompanied by limited surgical outcomes, for example, because of pigment deficiencies and consequently less sufficient laser coagulation responses.

The axial length of the eye can become the critical factor for reattaching a detached retina. For our retrospective and prospective patients, we selected a classification based on the literature in which pathologically myopic eyes with −6 dptr or higher negative values were distinguished from myopic eyes (between ≥−0.5 dptr and ≤−6 dptr), emmetropic eyes (between −0.5 dptr and +0.5 dptr), and hyperopic eyes (≥+0.5 dptr.).

While the proportion of pathologically myopic eyes increased from retrospectively 12% to prospectively 18%, proportion of myopic eyes from almost 19% to 37%, number of emmetropic eyes fell from retrospective over 36% to a prospective 32%, and the proportion of hyperopic eyes decreased from around 25% to less than 8%. In both groups, no clear data was available in some eyes, and so that proportion was reduced by a retrospective 8% to a prospective 5%.

In total, there was an increase in the overall risk due to significant shift in the direction of the myopic axis length with *p* = 7 × 10^−4^.

### 3.2.16. Retinal Hole: Total Area

According to their clinical importance, the retinal area and location were weighted into 6 categories. Retrospectively, 27 eyes (26.73%, category 6) did not have any holes or tiny defects preoperatively invisible behind the vitreous body.

A single hole could be presented preoperatively or intraoperatively at just one-third of the interventions (32 eyes/31.68%, category 5), 2 holes in 12 eyes (11.88%, category 4), 2 to 5 holes in 9 eyes (8.92%, category 3), more than 5 holes in 8 eyes (7.92%, category 2), and a giant tear or retinal detachment with macular hole in 13 eyes (12.87%, category 1).

Prospectively, 11 eyes (10.69%) were found in category 6, 22 eyes (21.36%) in category 3, 17 eyes (16.5%) in category 4, 22 eyes (21.36%) in category 3, along with 15 eyes (14.56%) in category 2, and as many in category 1. There was no data available for an intervention (0.97%). The risk profile shifts significantly (*p* = 0.004) with respect to the retinal surface area to “disadvantage” the prospective group. The prospective eyes have a significantly higher risk, especially in the case of risk assessments 2, 3, and 4 (2 to >5 holes, 29% retrospectively versus 53% prospective), whereas only a few eyes in the prospective group have only one or no holes (categories 5 and 6).

### 3.2.17. Retino/Retinectomies

Small cuts (retinotomies) lead to relaxation of smaller retinal contractions. If larger areas cannot be relaxed by retinotomies, the retina has to be removed (retinectomies).

In the case of a need of retinectomies (removal of a contracted, shortened, functional retina), categories were again created and intended to reflect the clinical risk profile:

- Category 1: retinectomy 360°
- Category 2: retinectomy 90–180°, 2 retinotomies posterior of the equator
- Category 3: retinectomy <90°, 1 retinotomy posterior of the equator
- Category 4: 2 retinotomies peripheral to the equator
- Category 5: 1 retinotomy peripherally of the equator
- Category 6: no retinectomy performed.

Retrospectively as well as prospectively, 360° retinectomy was not seen in either of the eyes (0% in category 1). 7 (6.93%) versus 5 (4.85%) eyes were classified retrospectively versus prospectively in category 2, 18 (17.82%) versus 16 (15.53%) met the criteria in category 3, 3 (2.97%) versus 0 (0%) met the criteria in category 4, 15 (14.85%) versus 8 (7.76%) met the criteria in category 5, and 58 (57.43%) versus 74 (71.84%) eyes were not retinectomized. With *p* = 0.11, there was no statistically significant difference.

### 3.3. Reduction of the Failure Rate as a Function of the Surgical Experience (Independent of Simulator Training)

A statistically significant difference (*p* = 0.002) between the retrospective and prospective study was found in the χ^2^ contingency panel test between the retrospective and prospective study, and the following survey was carried out to determine whether our main goal parameter “failure rate by 50% lower” also applies to the individual operating subgroups with different experience spectra.

This was also checked by means of a binomial test for the comparison of two Poisson frequencies by Fisher's F distribution with a rejection range *p*=2^∗^ (p/2).

As shown above, the retrospective failure rate of 35.64% (consisting of redetachment and duration fill) of the whole group was prospectively reduced (marginally relevant difference of delta0 = −0.1782) down to 6.8% with a *p* = 0.002.

Regarding the group of inexperienced surgeons (1 to 7 years of experience), retrospectively, a failure rate of 32.65% was found (Table 2). This resulted in a prospectively minimally relevant difference of delta0 = −0.1633. With a prospective failure rate of 4%, the target could be reduced by 50% with the subgroup of inexperienced surgeons (1 to 7 years) with double-sided questioning with *p* = 0.136.

In the case of the “average” surgeons (>7 years of experience), a retrospective failure rate of 47.22% differed from a prospective rate of 9.26%. This was shown as a highly significant difference by a two-sided test with *p* = 0.012.

The very experienced surgeons (>25 years of experience) retrospectively ended up with a failure rate of 18.75%, prospectively 4.17%. The null hypothesis could not be rejected with statistic significance (*p* = 0.655) in two-sided questions.

### 3.4. Effect of the Simulation Training on the Actual Operating Performance Depending on Surgical Experience

Whether simulator training just improves surgical confidence or also affects the actual surgical performance was a controversially discussed issue at the beginning of the study at hand. It had to made measurable based on criteria as anatomical success rate, surgical time, and so forth.

The influence of simulation training on the operative performance was obtained by means of the Eyesi surgical simulator. In Deuchler et al. [48], our group related the vitreoretinal surgery performance in Eyesi to that in the operating room and proved a statistical significant effect of VR warmup training to the actual service in the patients' eye.

Our study also showed that the expert typically caused less tissue damage, and score deductions had been limited to “time” and “completeness of the training module.”

In the following, we focussed on the deviation from the mean performance value depending on surgical experience and Eyesi training.

### 3.4.1. Analysis of Total Performance (with and without Warmup Training)

All video recordings (assessment scale 1–5) of the VR-to-OR performance (with and without warmup training, *n* = 21) of 4 surgeons who participated selectively in the Eyesi training module were evaluated by two independent observers: The surgeon with 25 years of experience (*n* = 7) achieved an average of 4.60 ± 0.21, while the other three participating surgeons with 2, 3, and 7 years of experience (*n* = 14) had mean values of 3.42 ± 0.76. Regardless of whether they had warmed up or not, the standard deviation of the 3 less experienced surgeons is very high compared to that of the expert with a SD = 0.76 (less experienced surgeons, *n* = 14) versus SD = 0.21 (expert, *n* = 7).

It was shown using the *F*-test for the comparison of two variances [41] in double-sided questioning with *p* = 0.005 that the variance *s*^2^ = 0.0441 of evaluations with the most experienced surgeon is significantly lower than with evaluations of the other participating surgeons with 2, 3, and 7 years of surgical experience with the spread *s*^2^ = 0.5776.

### 3.4.2. Analysis of Video Recordings without “Warmup” Training

The most experienced surgeon was evaluated with a score of 4.48 ± 0.07 (*n* = 5) without “warmup” training in real surgery. For the two surgeons with 3 and 7 years experience (*n* = 7), the mean evaluation score is 3.01 ± 0.54. The surgeon with 2 years of surgical experience was randomized with all his operations “with simulator training” (*n* = 2) and could, therefore, only be evaluated in this subgroup analysis. Using the *F*-test to compare two variances, the variance *s*^2^ = 0.0049 of evaluations in the most experienced surgeon was significantly lower than in evaluations of the other participating surgeons with 3 and 7 years of experience with the spread *s*^2^ = 0.2916.

### 3.4.3. Subgroup Analysis of the Video Recordings with “Warmup” Training

The most experienced surgeon (25 years of experience, *n* = 2 operations) has a mean score of 4.91 ± 0.04 and 3.83 ± 0.74 in the group of surgeons with 2, 3, and 7 years of experience (*n* = 7 operations).

Here, despite the spread of values, *s*^2^ = 0.0016 in the scores of the most experienced surgeon versus *s*^2^ = 0.5476 in the scores of the other participating surgeons with 2, 3, and 7 years of experience almost no significant difference was achieved in performing the *F*-test for the comparison of two variances in a two-sided questionnaire with *p* = 0.083. This is most likely explained by the low number of cases.

It can be clearly seen that the surgeon with 25 years of experience with (*s*^2^ = 0.0016) and without (*s*^2^ = 0.0049) warmup training has only minimal scattering in performance (Table 3). However, the scatter is much higher in the group of inexperienced surgeons.

Looking at the performance of surgeons separately, it was shown that warmup training resulted in a significantly better outcome of evaluations, but the spread of each inexperienced surgeon increased from 0.2916 (without warmup) to 0.5476 (with warmup). This is most likely explained by the fact that the total number is small and the most inexperienced surgeon with the highest standard deviation was randomized to the “warmup group” exclusively.

This shows that warmup training does not alter the performance scatter of the surgeons but the performance level of the surgeons.

### 3.5. Multiple Regression to Analyse Potential Risk Factors

In order to determine the most important influence factors for retinal redetachment in the retrospective versus prospective group (Table 4), we performed a multiple regression analysis. In this model, the statistical significance niveau is traditionally set at 10%. By combining the most important variables, multiple regression techniques show the overall explanatory power of the variables.

After performing multiple regression analysis with retinal redetachment rate as dependent variable, we could show that the most influential factor for retinal redetachment in the retrospective group was the amount of retino/retinectomies with *p* < 10^−4^ and the quality of documentation with *p* = 0.017.

After repeating the multiple regression with the same influence factors for the prospective group, all parameters were eliminated except for the amount of retino/retinectomies (Table 5).

The elimination of the constant “quality of documentation” in the prospective group was possibly achieved by our definition of standard operation procedures and evaluation protocols that improved consistency and information transfer between the different surgeons of primary and revision surgery. The surgeon, who took over the revision surgery, could control and if necessary, treat certain “weak” retina structures. The amount of retino/retinectomies plays the most crucial role for the outcome of retinal redetachment surgery with *p* < 10^−4^.

Both multiple regression analyses show a late elimination of the constant “timing of cataract surgery.” This was evaluated in detail in the following. Furthermore, we had to forgo the constant “kind of revision” in the prospective group, because we had to perform 100% 3-port revision surgeries instead of simple 2-port silicone oil removals.

Because of the improved documentation, the multiple regression analysis was repeated for further potential risk factors (Table 6) for retinal redetachment rate in the prospective group. These risk factors were not documented properly retrospectively but should not be neglected according to literature [49, 50].

Although fifteen instead of nine predictors were used, the same risk factors as before remained: the combination of the amount of retino/retinectomies with *p* = 0.003, the amount of membrane peeling with *p* = 0.028, and the timing of cataract surgery with *p* = 0.099 (both were eliminated late before). It indicates that pseudophakic patients or those being operated on the retina and cataract simultaneously less often get a retinal redetachment compared to patients who underwent cataract surgery during revision surgery or afterwards. Furthermore, the prognosis of eyes with retinal detachment surgery is better when a complete vitrectomy with posterior vitreous detachment and epiretinal membrane peeling is carried out, as well as ILM peeling if needed.

### 3.6. Influence of the Prospective Approach on Postoperative Vision

Apart from the significant reduction of the retinal redetachment rate to 6.80%, the prospective group also got a significant improvement in visual acuity with *p* < 10^−6^ using Mann–Whitney *U* test with a probability of *p* {(*X*|group1) < (*Y*|group2)} = 0.722565. Patients in the prospective protocol had a better vision outcome in 72.26%, which is important because after a successful retinal reattachment, the postoperative visual function is essential for patients. It certified that a primary reattachment is essential for a gain in vision. The exact correlation of these two parameters can be found below.

To find out if similar influence factors play a role in retinal redetachment as well as for postoperative vision, we repeated the multiple regression analysis with the dependent variable postoperative vision (Table 7).

The results show that besides a low P(D)VR stage (*p* = 0.001) and a good preoperative fovea situation (*p* = 0.074), a permanent attachment of the retina (*p* = 0.041) leads to a better prognosis of postoperative vision. Furthermore, the primary and revision surgery should remain in one surgeon's hands (*p* = 0.089).

### 3.7. Correlations between Risk Factors in the Prospective Study Part

After finding the most important risk factors for retinal detachment and follow-up of vision, we looked for correlations between these individual factors. In the following, the significant correlations are listed.

### 3.7.1. Factors Associated with the Best Postoperative Visual Acuity

The visual acuity is the most widely used form of performance testing, for example, the reattachment of a formerly detached retina. However, it is by no means suitable to testify actual visual quality. Therefore, a contrast vision test is also requested in the evaluation list for checking the quality of vision.

The detection of visual quality with the Vimetrics® Central Vision Analyser (CVA) impressively enables the evaluation of visual function under the conditions of a retinal detachment with and without macular involvement, before and after retinal reattachment surgery under mesopic and photopic conditions. This helps to determine why the affected person is a subject to other visual limitations than those to which the pure visual test implies.

*(1) Best Postoperative Visual Acuity/Course of Visual Development/Retinal Attachment*. A Spearman [34, 51] correlation coefficient of rho = 0.26 and a statistical significance of *p* = 0.006 was found for visual acuity gain with retinal attachment, and a correlation coefficient of rho = 0.29 with *p* = 0.003 for retinal attachment and the best postoperative visual acuity.

*(2) Best Postoperative Visual Acuity/Preoperative Situtation/Intraoperative Course*. Foveal attachment (*p* = 0.006, rho = 0.27) and a low degree of proliferation (P(D)VR stage) were associated with better visual acuity (*p* = 4 × 10^−4^, rho = 0.35). Patients with a better postoperative visual outcome (*p* = 7 × 10^−6^ and rho = 0.42) also showed more vision gain during the course of the study. If the intraoperative risk profile was low, this initial situation also correlated with better results for the visual acuity (*p* = 0.01, rho = 0.23).

*(3) Best Postoperative Visual Acuity/Surgeon Consistency.* “Everything in one surgeon's hand” leads to better visual results (*p* = 0.04, rho = 0.20). This is not as trivial as it sounds, because all patients are evaluated in a team of supervisors, surgeons, and assistants.

*(4) Best Postoperative Visual Acuity/Gain of Visual Acuity/OCT*. There was a highly significant correlation between the best visual acuity and the morphometry measured with the optical coherence tomography (OCT), (*p* < 10^−6^, rho = 0.57). Correspondingly, the visual acuity gain correlated with the OCT result with *p* = 0.026 and rho = 0.22.

*(5) Best Postoperative Visual Acuity/Contrast Vision/Oil Type/Retina Attachment*. In principle, 5000 mPa·s and 4300 mPa·s silicone oils had more stability than those with 2000 mPa·s. Differences were not significant, neither regarding the retinal reattachment rate (*p* = 0.68) nor the visual acuity (*p* = 0.33) or the contrast vision (*p* = 0.34).

*(6) Best Postoperative Visual Acuity/Contrast Vision/OCT/Fovea Attachment/Risk Profile/P(D)VR/Autofluorescence*. Between the best visual acuity and contrast vision, there was a highly significant correlation with *p* < 10^−6^ and rho = 0.62. The seemingly trivial nature of this is discussed.

Depending on the actual situation immediately before retinal detachment surgery, an even more important aspect emerges in that the actual visual acuity gain is based on the operative care. There is a significant correlation between visual acuity and contrast visual gain (*p* = 0.04, rho of 0.22).

A lower intraoperative risk profile correlates with better visual acuity (*p* = 0.01, rho = 0.23). A homogeneous autofluorescence correlated significantly with a better postoperative visual acuity (*p* = 9 × 10^−6^, rho = 0.436) as well as the contrast vision (*p* = 2 × 10^−6^, rho = 0.483) and a good OCT result (*p* = 3.8 × 10^−5^, rho = 0.406).

### 3.7.2. Factors Not in Context with Visual Acuity

*(1) Increase in Contrast Vision/OCT-Assessment/Autofluorescence/Perioperative Course*. There was a statistically highly significant correlation between contrast and OCT with *p* = 3 × 10^−6^. The better the clinical contrast that the patient developed during the course of the study, the more homogeneous the structure of the eye with a correlation coefficient of rho = 0.465 in the last visit—with a good receptor layer thickness without swelling or atrophy in the OCT. If the fovea was present during primary retinal detachment surgery, the contrast vision was better (*p* = 0.046, rho = 0.206).

The lower the P(D)VR activity, the more favorable the contrast vision development (*p* = 0.04, rho = 0.21), structural preservation of the retina in OCT (*p* = 0.003, rho = 0.29), and its autofluorescence (*p* = 0.09, rho = 0.17).

Moreover, the lower the intraoperative risk profile (*p* = 0.018, rho = 0.237), the more homogeneous the autofluorescence.

*(2) Retinal Attachment/Time of Cataract Surgery*. When a correlation between cataract surgery timing and retinal detachment was performed, a clear trend with *p* = 0.06 showed that the earlier a cataract operation was performed (preoperatively or at least during oil removal), the better the success rate of the retinal detachment (rho = 0.188).

This verifies the results of Fisher-Freeman-Halton's exact contingency panel test [52], comparing the failure rate between the preoperative pseudophakic versus phakic group; a higher success rate among the already pseudophakic patients with a *p* value with Valz and Thompson's algorithm of *p* = 0.039.

*(3) Degree of Laser Coagulation/Emulsification Rate/P(D)VR Activity*. In Spearman's rank correlation, a significantly negative correlation between laser quality (high-scale value = high number laser spots) and emulsification rate (high-scale value = low-emulsification rate) occurred (*p* = 0.03, rho = −0.21). Additional laser was performed once oil filled into the vitreous cavity induces a higher emulsification tendency.

A low rate of emulsification (evaluated by blisters in the VB, AC, or lens capsule) correlated negatively (*p* = 0.018, rho = −0.24) with a more advanced P(D)VR stage. At first glance, this confusing result will be discussed (advancement of severity P(D)VR versus inflammatory activity and emulsification rate).

*(4) Documentation/Degree of Laser Coagulation/Intraoperative Risk Profile*. The quality of the documentation of the procedure and that of the laser coagulation correlate with a *p* value = 0.01 and rho = 0.24. If a lot of laser spots have been applied, it was very well documented. The diligence of the documentation was requested by the SOP, with laser documentation being provided in both text form and graphically.

With a clear trend, the quality of the documentation was improved (*p* = 0.059, rho = −0.186) with increasing risk profile (more retino/retinectomies), such as intraoperative insight into the severity and complication of the intervention.

*(5) Intraoperative Risk Profile/Severity Degree P(D)VR/Retinal Redetachment*. There was a highly significant correlation between intraoperative risk profile and severity of the P(D)VR staging (*p* = 9 × 10^−4^, rho = 0.322). This can be explained by the membrane-induced stiffening of the retina with the consecutive tediousness of tissue removal (retinotomy/retinectomy). The more severe the P(D)VR and the higher the intraoperative risk profile (*p* = 0.005 and rho = 0.33), the greater the risk of redetachment of the retina (*p* = 0.016, rho = 0.237).

*(6) Patient Age/Eye Disease/Membrane Peeling*. When retinal detachment occurred, more severe eye changes were seen in younger patients (*p* = 0.017, rho = 0.235), for example, in uveitis intermedia or infectious eye inflammation (e.g., HIV). Also in younger patients, significantly more membrane peeling (*p* = 0.02, rho = −0.22) was necessary.

*(7) Membrane Peeling/Retina Holes*. The higher the number and extent of the retina holes in one eye (*p* = 0.057, rho = 0.188), the more intense an ERM and ILM peeling was required.

### 3.8. Analysis of Interactions between Silicone Oil and Eye Tissue

#### 3.8.1. Results of Microscopic Oil Analysis in the Laboratory

In Deuchler et al. [52, 53], the focus was on potential connections between different types of silicone oils and their emulsification characteristics [54–60]. As presented, the measurements of the oils were taken with the aid of a Bresser-Trino research field microscope (with the help of alamedics GmbH & Co.KG, Dornstadt, Germany) showing very different distribution densities (0–250, 250–500, 500–750, 750–1000, and >1000 oil bubbles/cm^2)^.

Different bubble diameters are graphically represented here.

**Figure 3:**
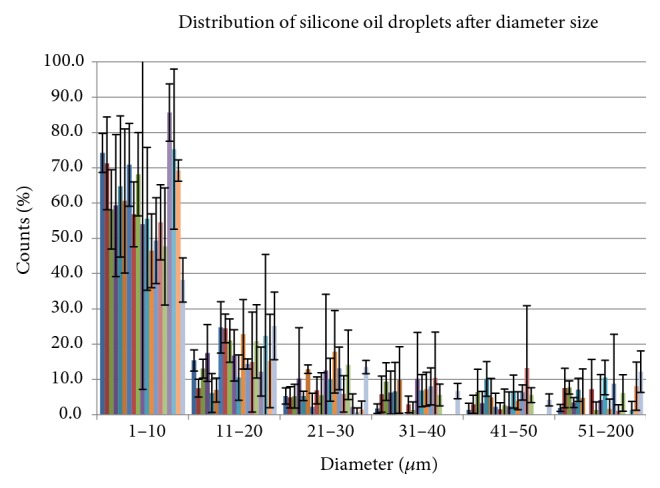
Distribution of silicone oil droplets after diameter size, in % with standard deviation.

With the percentage distribution of the emulsification bubbles by their diameter, it was found that the majority of the emulsification bubbles have a diameter of only 1–10 *μ*m (Figure 3). This greatly increases the risk that emulsification bubbles enter the chamber angle and could potentially lead to secondary glaucoma, justifying our decision for a 3-port oil removal revision surgery in which the vitreous space can be repetitively rinsed.

In the context of the subgroup analysis of completely evaluable samples from 19 eyes, the connection between the type and extent of emulsification and preceding anterior segment operation or retinal preoperations before oil removal (oil analysis) is emphasized, as well as the connection between emulsification, failure rate, and the amount of laser coagulation not before but after filling the silicone oil into the glass body.

#### 3.8.2. Effect of Silicone Oil on the Lens Maturation (Scheimpflug Examination with Pentacam®)

In Deuchler et al. [52, 53], we published important correlations between the lens status and the success of retinal reattachment surgery with temporary silicone oil fill.

When analysing the lens transparency loss projecting the reference body through the whole lens, the Wilcoxon test for four age groups (group 1 (mean 69.5 years), group 2 (mean 62 years), group 3 (mean 51 years), and group 4 (mean 36 years) (*p* < 0.05)) proved for all except for the younger ages with significant lens changes (*p* ≤ 0.05). The strongest signs of maturation were obvious in the age groups 2 and 3.

When analyzing the sections of the lenses separately, the lenses in group 3 showed significant transparency changes in all parts, and in group 2, changes in the anterior and central part were significant (*p* = 0.004), but not in the posterior part (*p* = 0.375). The lens sections separately analysed showed no significant changes in the oldest group (group1), and most pronounced changes here were found in the anterior part (*p* = 0.094).

Obviously, the lens transparency loss plotted against age does not show a linear function: the lens transparency loss increases from group 4 (youngest group) through group 3 to group 2 but decreases in the oldest group 1. In Deuchler et al. [52], the relationships between temporary silicone oil filling and the individual lens change when performing a PPV with temporary silicon oil filling to manage a complicated retinal detachment was discussed in detail. It was speculated that older patients (group 1) already had a relatively more advanced cataract before surgery with silicone oil instillation.

#### 3.9. EVALP/SOP: Consequences of the Statistical Findings for the Protocols

The current retrospective data record shows the role of insufficiently accurate documentation of individual parameters: for example, the quality of laser coagulation should be specified for individual segments of the retina separately. In the case of an insufficient primary response to laser applied primarily and no proper additional laser where necessary (during revision surgery or in between), redetachments of the retina after silicone oil removal are likely. A better documentation can compensate the negative effect of the visual outcome if more than one surgeon has operated on one eye.

The adaptation of an evaluation protocol and the development of SOPs were a common thread throughout the entire study [53]. It was clear from the beginning that both would be a prerequisite for successful vitreoretinal surgery and the treatment of complicated retinal detachments. The necessary data set was immediately transferred postoperatively to the developed evaluation sheets. For creation of the 6-sided evaluation protocol, known parameters from the literature (e.g., P(D)VR stage and amount of retinectomy) were used as well as parameters which were significantly important in the retrospective analysis (e.g., amount of membrane peeling and quality of laser coagulation).

The careful creation of the initial EVALP took 3 months, and its format was continually revised and adapted during the study. At the end of the study, the final version was optimized in correlation to the ergonomics. The EVALP was finally revised in such a way that this form of evaluation should take, on average, about 10 minutes to complete for each operating unit. Therefore, it can be recommended to the general public as a quality assessment sheet.

The SOP at the end of the study shows two major content-related changes compared to the initial version: the option of removing silicone oil “passively” via a 2-port access from the eye no longer exists because of changes in the manufacturing process. Furthermore, we have to reduce the silicone tamponade time from 4 to 2 months.

Firstly, our analysis shows that a redetachment usually occurs within 4 weeks after oil removal; secondly, it is still an open question to what extend retinal damages can be provoked by long standing silicone oil fill.

## 4. Discussion

Even if the group of eyes followed up retrospectively versus prospectively were expected to show lots of differences, essential baseline and demographic parameters proved to be homogeneous: gender, distribution of the right and left eyes, fovea involvement, efforts to laser retina or peel membranes, and, last but not the least, there did not exist any noteworthy differences regarding the staging of PVR or PDR (=P(D)VR).

Parameters like “amount of membrane peeling” and “revision strategy (2-port/3-port access)” had changed due to the introduction of our SOP.

That the parameter “axial lengths” shifted more towards a higher degree of myopia (from retrospective towards prospective) underlines our improved retinal reattachments rate because higher myopia in general is expected to have a negative impact onto the success rate.

After analyzing the subgroups with different surgical experience, we found an improved success rate in all groups. The most pronounced effect could be observed in the group with intermediate experience. Obviously SOPs and EVALPS are most effective when a surgeon has overcome starting issues but has not reached expert levels.

The rate of complication “redetachment of the retina after PPV with a temporary silicone oil tamponade” was reduced from about 35% to approx. 7%.

Only the eyes, which after the final removal of silicone oil had a permanent retinal attachment, were classified a success and differentiated from those redetaching and/or requiring a permanent oil fill to stabilize a partial or complete retinal attachment.

Eyes with partially or completely redetached retinas and those permanently filled with oil were considered to be a failure, since the patient does not experience a final morphological or functional rehabilitation.

This result is only helpful if the cause analysis leads to significant explanatory possibilities that allow reproducible results for all. Since the introduction of the 3-port PPV technology by Machemer et al. [61], this PPV procedure has allowed to precisely remove the traction component in the environment of a retinal hole. Meanwhile, there are sufficient long-term observations on representative cases [62–65].

Various authors refer to strong fluctuations in retinal redetachment rates between <10% and >70%.

Whoever tries to analyse the given data from other groups and to compare it with the own outcomes usually does not achieve a satisfactory result in the absence of SOPs and comparable EVALPs [66].

Choudhary et al. [13] have a correspondingly low-complication rate of just under 4% in the retrospective analysis of their pars plana vitrectomies with silicone oil tamponade for the management of so-called complicated retinal detachments.

In their series, retinal redetachments occurred within 6 weeks (in our series, within 4 weeks). Are there connections between their retrospective data collection and data in our study? What conclusions can be drawn, if any?

The measurement parameters “anatomical reattachment rate,” “best visual acuity,” and “intraocular pressure” appear to be essential but cannot sufficiently explain the overall good result.

An “aggressive approach” with vitrectomy in front of the outer vitreous base makes a lot of sense. In advanced P(D)VR, the need for retinotomies and retinectomies in the outermost periphery is very likely [13, 67]. Where tractions prevent retinal reattachment, the removal of tissue (including retinal tissue) is inevitable in many cases.

An “as complete as possible filling” of the vitreous cavity with silicone oil is an important issue often mentioned in literature, but difficult to measure and hardly comparable between different investigations. Argon laser coagulation is also one of those obviously necessary parameters which should have a positive effect on the outcome. In the study by Choudhary et al. [13], this is achieved in all cases as a 360° laser coagulation. However, one question remains unanswered: What stage of P(D)VR did the authors have to deal with? How extensive were the retinotomies/retinectomies been performed?

The following statements were made: 167 of 173 (96.5%) of the eyes were classified as anatomically successful.

The mean length of stay of the oils was 70 ± 48 weeks, which is significantly higher than in our study. 21% P(D)VR versus 79% PVR are similar to our ratios of 14.6% P(D)VR versus 85.4% PVR. The best visual acuity [13] was 0.2 or higher in 50% of all eyes 3 months after oil removal; in our study, there was no patient completely blind (no light perception); 4 months after oil removal, 10% of patients had a visual acuity between light perception, and 1/50 and a quarter of the patients (26.2%) had visual acuity between 1/50 and 0.1, one third of the patients (34.9%) between 0.1 and 0.3, 17.5% developed vision 0.3 to ≤0.5, and 10.7% saw better than 0.5; in more than a quarter of our patients, no visual aids were needed for reading (visual acuity of at least 0.3).

Chaudhary's working group was able to remove the oils in all cases via a 2-channel access, and postoperative “floaters” (oil bubbles) in almost 10% of all patients seemed tolerable and did not lead to any further complications—if one disregards a potential association with a 7.5% secondary glaucoma rate which required treatment with cyclocryocoagulation. In our prospective work (oil drainage via 3-port access including rinsing of residual oil bubbles), there was no secondary glaucoma. Indeed, we had to flush the vitreous space at least once after removing the main oil bubble to completely remove satellite bubbles which are best accessible during repeated fluid air exchange.

Although Chaudhary's group mentions retino/retinectomies as parameters which are indicated when membrane remedies cannot be removed from the retina, the extent of these tissue removals is not explained in detail.

Prognostic factors for the long-term reattachment of the retina and a positive visual outcome are discussed in various contradictory ways. In principle, Grigoropoulos et al. [68] consider a good visual result to be possible even with heavier pathologies and the possible need for multiple, larger retinectomies.

A smoldering P(D)VR as well as hypotonia is prognostically unfavorable factors, whereas a shorter oil tamponade, the oil removal itself, small retinectomies, only few preoperative surgical interventions, and a good visual acuity are favorable factors as a starting point. It is important to “limit the number/size of retinectomies, but to retinectomize in time.”

In our group, we tried to define the requirements for the adequate retinectomy issue as follows: in the areas where the vitreous exerts traction in proximity to a retinal hole, either the tractions must be completely removable or the retinal detachments must be extended until the contracted tissue is stress-free attachable to the underlying choroid RPE complex. The success of this procedure must not only be judged under air or heavy liquid (forced preliminarily) but must also be ensured under physiological fluid (as BSS).

A basic consideration should be as follows: to what extent is the staging of the P(D)VR and/or its smoldering activity, de facto decisive for the outcome prognosis? We do know from extensive research carried out by the group of Charteris: if at all, a combination of fluorouracil and heparin exclusively in the early stages of P(D)VR can (prophylactically) influence this most disastrous event [47, 69]. Decent information which could contribute to a better understanding of this disease can probably only be gained if the removed vitreous is systematically examined for VEGFs and interleukins. Obvious is that the risk for a permanent retinal detachments correlates with the amount of retinectomies and the efforts which have to be taken to repair the retinal detachment by removing retinal tissue can also contribute to further proliferative vitreoretinopathy activity.

De Silva et al. [70] focus on the importance of the etiology of the retinal detachment and on the relevance of the P(D)VR staging, and a 360° laser coagulation considered to be a positive predictor for the anatomical and functional result.

We know that laser coagulation of the freshly applied retina might not be able to be carried out in an equally efficiently and in a low-risk manner (retinal swelling, leakage risk) in many areas; additional coagulation has eventually to be carried out in the free interval between the first and second interventions or during revision surgery later on.

In De Silva's working group [70], adequately performed laser coagulation reduces the retinal redetachment rate from 58% to 26% after silicone oil removal.

Our results supplement these observations in regard to the functional success rate (postoperative visual gain). Here, the visual gain increases with the number of laser coagulation spots. Extensive laser coagulation during the primary supply (final silicone oil filling) has no negative effect on visual acuity and visual quality [71, 72]. However, during revision surgery (silicone oil removal), an additional laser coagulation should be limited to the absolutely necessary diagnostic/therapeutic need. Surgical time and effort, timing of a cataract surgery in view of specific lens changes under silicone oil tamponade [52, 53, 73], torsion instability of thinner instruments, so-called flow dynamics (“fluidics”), estimation of inflammation parameters in the vitreous space, and so forth are only some of the parameters that in the future will have to be worked up from these points of views, especially since we have gained negative experience with preoperative, incompletely vitrectomized eyes within the framework of this work.

Without doubt, the early entry (<4 weeks) into an eye with persistent vitreous hemorrhages has to be preferred due to the reduction of proliferative activities (P(D)VR); however, the supposed “attractiveness” of smaller gauges and the associated need for modification of the procedure should not lead to a “small”, incomplete removal of the vitreous when P(D)VR requires a meticulous procedure in the outer vitreous periphery as well as peeling of epiretinal membranes in the macula [74].

Although a “total” removal of the vitreous body cannot be attained purely mechanically, the removal of any vitreous which exhibits tractive effects on the retina during the intervention or at the end of the procedure immediately before the silicone oil filling has to be carried out as completely as possible. Otherwise, it serves as a recruitment area for P(D)VR activity processes. This should be kept in mind when applying low-gauge instruments—although sufficient for removal of an uncomplicated macula pucker or a macula hole, these instruments may not be able to fulfill the requirements for cumbersome removal of pathologically changed vitreous in P(D)VR.

## 5. Conclusions/Recommendations

Our recommendations for future approaches that we like to sum up here are mainly based on not only the key factors discussed above but also other practical findings with effect onto the outcome of surgery:

- We generally recommend using a defined treatment path (SOP) and an EVALP, which should promptly and carefully be considered by all medical staff in attendance. The protocols prepared and revised in the present analysis can serve as a guide for other operating units. According to our literature research, there is no consensus about SOPs and EVALPs which would allow to relate procedures and outcomes of different centers to each other. Only a uniform approach, according to SOPs and EVALPs, make protocol controlled procedures and their results comparable.
- Preferably, in all required interventions, the service for a patient's eye should be provided by one and not by different surgeons because of the ultimately demonstrable positive effect on the development of visual acuity. Once you apply an EVALP and SOP, case- and situation-related exceptions from the “one-surgeon-only-service” are conceivable. Protocolled details about the course of disease and surgery allow a 2nd and 3rd surgeon to improve further steps of treatment of the same eye. Essentially for the effectiveness and safety of the service in a vitreoretinal center also should the option for any surgeon to make use of a more experienced colleague to assist the case or even take over from the less experienced surgeon.
- Once silicone oil has been removed, the follow-ups of the operated eye must be carefully structured during the first four weeks, including the instructions for the patient about how to perform self-monitoring.
- If triamcinolone or ICG-assisted dusting or staining of the vitreoretinal interface demonstrates clinical relevant pathological changes, a peeling of the epiretinal tissue with or without peeling of the ILM should be performed. The nature and extent of peeling-associated retinal hemorrhages suggest to continue peeling, starting from the vascular arcades, working towards the fovea with reduced speed.
- The vitreous body must be removed as carefully and completely as possible in the outermost retinal periphery of the present pathologies with tissue proliferation. For this purpose, the choice of the surgical procedure (one-handed, bimanual) and the necessary tools (hand-held light pipe, chandelier, and so forth) must be weighed for this procedure as well as a decision on the status of the eye lens. If desired, a defined amount of the triamcinolone typically used to detect residual vitreous can be left in the eye as a potentially anti-inflammatory agent.
- Due to the particular challenge involved in the treatment of proliferative vitreoretinopathies, instruments and optics should be tailored to both pathological lengths of the eye as well as special requirements in the context of decreasing gauges (instrument design, instrument stability, reduction lenses for different observation systems, light sources, and light guides). To date, the use of 27 g instruments should be reserved for the treatment of pathologies without heavy secondary response to inflammatory activity.
- The use of PFCL has to be weighed critically. In principle, the heavy liquids and silicone oils available on the market since around 2012 come with more emulsification side effects than those from many years before. Without retinal cracks, the retina can also be reattached via a liquid-air drainage without temporary PFCL instillation. If the use of PFCL in the vitreous cavity is necessary, preferably the heavy liquid should not be filled up beyond the equator of the eyeball, because remnants of this substance can easily stick to the ciliary body tissue. Of the two options, PFCL-silicone oil-direct exchange and PFCL-via air-to-silicone oil-exchange, the latter one should be the preferred for a thorough removal of the PFCL.
- Due to the increasing silicone oil emulsification rate over time, we have now concluded that silicone oil should be preferably removed already after 2 months. This might also help to lower the rate of “visual loss for unknown reasons after temporary silicone oil tamponade.” With regard to the final structural outcome, the postoperative visual acuity, or the rate of emulsification, no recommendations for a certain oil or special viscosity can be given.
- Other factors affecting the type and extent of emulsification include the strategy of oil instillation as well as the “timing” of laser (under BSS, air, and oil) when injecting silicone oil through cannulas with small gauges, speed has to be reduced to the threshold where air bubbles will not build up, and laser, in general, should be avoided or limited once oil is filled into the vitreous cavity.
- It is necessary to define circumstances when the surgeon—regardless of his experience—has to recall his skill level in the Eyesi and/or run through individually tailored “warmup programs” before providing service in the operating room. Particularly for colleagues “in-training,” a short curriculum must be drawn up so that a preoperative training on Eyesi is possible thus decreasing the risk of causing complications.
- Emulsification management including strategies to remove “sticky oil” as well as the management of vitreoretinal diseases in eyes with pathological axis length should be integrated into Eyesi and be trainable.
- It has to be prospectively established when and under which criteria the lens should be operated on, either before or simultaneously with the primary retinal reattachment including silicone oil instillation. As much as leaving the lens in place can have a stabilizing effect when the eye suffers from inflammatory diseases, the lens itself can be “in the way” and make it difficult to allow access to the peripheral vitreous space. When delayed, opening the anterior lens capsule during cataract surgery might be significantly more difficult since after temporary silicone oil tamponade, the lens nucleus and all layers in front of it show significant changes. This affects particularly the middle age groups (50–70 years).
- The current classification(s) of a P(D)VR do not allow estimation of the actual ratios of endothelial/fibrotic activities for the different stages of P(D)VR (stages A until stages C/D). Since growth factors (VEGFs) and inflammatory mediators (interleukins) play an essential role in the emerging cascade of retinal detachments with P(D)VR, a concept has to be developed to measure these.
- As long as no significantly improved silicone oil products are offered, it will be necessary to choose a complete 3-port PPV strategy to reliably remove the silicone oils from the eye by repeated rinsing procedures. A modern illumination technique (extraocular, diaphanoscopically) could allow such a procedure to be performed in the future, choosing a 2-port PPV approach.
- The total surface area of all retinectomies should be kept as small as possible: enrolled edges of retinal holes, necrotic tissue, and dispersed pigment epithelial cells which have to be trimmed back, respectively, and removed from the globe as complete as possible (reduction of PVR potential).
- To evaluate the postoperative success, vision quality assessment tests that provide information for vision under various conditions (e.g., contrast and glare), visual acuity measurements, and OCT documentation of structural changes should be assigned to reflect subjective vision and give a more reliable prognosis for final visual outcome.
- Less experienced surgeons typically show significant performance fluctuations and benefit most from improved operational documentation and standardized procedures.
- Only a uniform approach, according to SOPs and EVALPs, makes protocol-controlled procedures and their results comparable.

## Figures and Tables

**Table 1:** Parameters of population and surgery (retrospective versus prospective) and their statistical relation.

Number of eyes/patients	101 (retro)	103 (pro)	*p* value
Age, years			0.0005^1^
Mean ± SD	63.35 ± 13.32	57.08 ± 11.81	
Range	26–101	22–86	
Gender, number			0.99^∗^^2^
Male	65	66	
Female	36	37
Eye, number			0.48^∗^^2^
Right	51	57	
Left	50	46
Preop. fovea situation			0.94^∗^^2^
Attached	21	22	
Washed up	15	18	
Detached	60	63
Not specified	5	0	
Redetachment rate			4 × 10^−6^∗2^^
None	65	96	
Permanent oil fill	4	4	
Redetachment ≥4 months	7	1	
Redetachment 1–4 months	4	1	
Redetachment <1 months	21	1	
Surgeon‘s skill, years			0.002^∗^^2^
1–7 years	49	25	
≥7 years	36	54	
≥25 years	16	24	
Timing of cataract surgery			0.06^∗^^2^
Primary pseudophakic	29	41	
With silicone oil fill	8	3	
With revision surgery	35	41	
In the course	29	18
P(D)VR stage			0.71^∗^^2^
AB/A	26	33	
C1/B	12	13	
C2/C1	18	16	
C3/C2	15	20	
D1/C3	9	14	
D2, D3/D1	11	7
Not specified	10	0	
Viscosity of silicone oil			0.01^∗^^2^
5000 mPa·s	80	69	
4300 mPa·s	4	6	
2000 mPa·s	13	28	
Not specified	4	0	
Laser coagulation (amount and efficiency)			0.11^∗^^2^
Grade 6	10	6	
Grade 5	27	30	
Grade 4	20	33	
Grade 3	21	24	
Grade 2	11	9	
Grade 1	7	1	
Not rated	5	0	
Amount of membrane peeling			< 10^−6^∗2^^
VB + PVD + ERM + ILM peeling	4	79	
VB + PVD + ERM peeling	22	11	
VB + PVD	72	12	
VB	3	1	
Consistency of surgeon			0.76^∗^^2^
In one hand	26	30	
2 surgeons	19	16	
>2 surgeons	55	57	
Not specified	1	0	
Kind of revision			10^−6^∗2^^
2-port	74	0	
3-port	23	103	
Not specified	4	0	
Peeling at the time of revision			0.82^∗^^2^
Performed	18	17	
Not performed	81	83	
Not specified	2	3	
Axial length			0.0007^∗^^2^
Patho. myopic	12	19	
Myopic	19	38	
Emmetropic	37	33	
Hyperopic	25	8	
Not specified	8	5	
Number and size of retinal hole area			0.004^∗^^2^
1 (macula hole, giant tear, 360° tear)	13	15	
2 (>5 holes, <10 hrs)	8	15	
3 (>2 < 5 holes, <8 hrs)	9	22	
4 (=2 holes, <6 hrs)	12	17	
5 (=1 hole, <4 hrs)	32	22	
6 (hidden hole, <2 hrs)	27	11	
Not specified	0	1	
Retino/retinectomy			0.11^∗^^2^
1 (retinectomy 360°)	0	0	
2 (retinectomy 90–180°, 2 retinot. post. to the equator)	7	5	
3 (retinectomy <90°, 1 retinot. post. to the equator)	18	16	
4 (2 retinot. ant. to the equator)	3	0	
5 (1 retinot. ant. to the equator)	15	8	
6 (not performed)	58	74	

Statistical analysis: ^1^two-sample *t*-test: ∗^2^χ^2^ contingency table. Number: number of patients; SD: standard deviation; mPa·s: millipascal·second; VB: vitreous body; PVD: posterior vitreous detachment; ERM: epiretinal membrane; ILM: membrana limitans interna; retinot.: retinotomy; post.: posterior; ant.: anterior; preop.: preoperative; patho.: pathological.

**Table 2:** Failure rate depending on surgical experience.

Surgical experience (years)	Retro: surgery (*n*)	Failure rate (*n*)	(%)	Goal (%)	Pro: surgery (*n*)	Failure rate (*n*)	(%)	Stat. signific. (*p*)
Overall	101	36	35.64	<17.82	103	7	6.80	0.002^∗^^1^
1–7 years	49	16	32.65	<16.33	25	1	4.00	0.136^∗^^1^
≥7 years	36	17	47.22	<23.61	54	5	9.26	0.012^∗^^1^
≥25 years	16	3	18.75	<9.38	24	1	4.17	0.655^∗^^1^

Statistical analysis: ^∗^^1^two-sided binomial test. *n*: number of surgeries.

**Table 3:** Performance scatter depending on surgical experience.

Surgical experience in years	Mean without warmup	SD without warmup	Mean with warmup	SD with warmup	Mean without + with warmup	SD without + with warmup
25	4.48	0.07	4.91	0.04	4.60	0.21
3	2.96	0.24	3.47	0.86	3.13	0.50
7	3.08	0.89	4.10	0.66	3.59	0.89
2	—	—	3.79	1.11	—	—
2, 3 + 7	3.01	0.54	3.83	0.74	3.42	0.76

SD: standard deviation.

**Table 4:** Multiple regression to analyse potential risk factors for retinal redetachment rate in the retrospective group.

Model		Coefficients^a^			*t*	Sig.
		Not standardized coefficients		Standardized coefficients		
		*B*	Standard deviation	Beta		
1	(Constant)	.096	1.573	—	.061	.951
	Emulsification rate	−.040	.097	−.042	−.412	.681
	Surgical experience	.059	.178	.031	.331	.741
	Timing of cataract surgery	.233	.196	.123	1.188	.238
	Med. history/ reop. surgery	−.013	.131	−.010	−.102	.919
	Amount of retino/retinectomies	.504	.125	.394	4.026	.000
	Quality of documentation	.653	.255	.264	2.556	.012
	Quality of laser coagulation	−.140	.144	−.095	−.973	.333
	Amount of membrane peeling	.097	.133	.070	.734	.465
	Consistency of surgeon	.128	.106	.114	1.203	.232
	Kind of revision	.041	.448	.009	.092	.927

9	(Constant)	1.007	.679	—	1.484	.141
	Amount of retino/retinectomies	.515	.115	.402	4.468	.000
	Documentation	.543	.223	.220	2.438	.017

^a^Dependent variable: retinal redetachment rate.

**Table 5:** Multiple regression to analyse potential risk factors for retinal redetachment rate in the prospective group: in this group, all patients got a 3-port revision surgery, so that “kind of revision” was not a predictor in this model.

Model		Coefficients^a^			*t*	Sig.
		Not standardized coefficients		Standardized coefficients		
		*B*	Standard deviation	Beta		
1	(Constant)	3.456	1.008	—	3.427	.001
	Emulsification rate	−.014	.062	−.021	−.218	.828
	Surgical experience	−.015	.128	−.014	−.118	.906
	Timing of cataract surgery	.169	.115	.165	1.474	.144
	Med. history/preop. surgery	−.008	.087	−.011	−.092	.927
	Amount of retino/retinectomies	.238	.069	.347	3.439	.001
	Quality of documentation	.092	.109	.086	844	.401
	Quality of laser coagulation	−.017	.118	−.015	−.143	.886
	Amount of membrane peeling	.062	.059	.105	1.054	.294
	Consistency of surgeon	.014	.068	.024	.205	.838

9	(Constant)	4.474	.331	—	13.524	.000
	Amount of retino/retinectomies	.245	.064	.356	3.831	.000

^a^Dependent variable: retinal redetachment rate.

**Table 6:** Multiple regression to analyse further potential risk factors for retinal redetachment rate in the prospective group.

Model		Coefficients^a^			*t*	Sig.
		Not standardized coefficients		Standardized coefficients		
		*B*	Standard deviation	Beta		
1	(constant)	4.461	.862	—	5.174	.000
	Emulsification rate	.006	.041	.016	.158	.875
	Surgical experience	.028	.082	.041	.345	.731
	Med. history/preop. surgery	.030	.051	.065	.581	.563
	Documentation	.068	.070	.103	.973	.333
	Quality of laser coagulation	−.003	.075	−.004	−.041	.968
	Amount of membrane peeling	.075	.038	.202	1.977	.051
	Consistency of surgeon	.016	.043	.044	.367	.715
	Timing of cataract surgery	.148	.070	.232	2.104	.038
	P(D)VR stage	.048	.058	.101	.822	.413
	Preop. fovea situation	.016	.042	.041	.373	.710
	Amount of retino/retinectomies	.107	.049	.251	2.201	.030
	Number and size of retinal hole area	.013	.053	.026	.243	.809
	Axial length	−.048	.047	−.108	−1.039	.302
	Peeling at the time of revision	−.286	.181	−.154	−1.581	.118
	Laser coagulation at the time of revision	−.266	.212	−.127	−1.255	.213

13	(Constant)	4.329	.366	—	11.830	.000
	Amount of membrane peeling	.078	.035	.210	2.237	.028
	Timing of cataract surgery	.099	.059	.155	1.667	.099
	Amount of retino/retinectomies	.123	.040	.289	3.091	.003

^a^Dependent variable: retinal redetachment rate.

**Table 7:** Multiple regression to analyse essential factors for good postoperative vision in the prospective group.

Model		Coefficients^a^			*t*	Sig.
		Not standardized coefficients		Standardized coefficients		
		*B*	Standard deviation	Beta		
1	(Constant)	.038	1.180		.032	.975
	Emulsification	.080	.057	.139	1.402	.165
	Surgical experience	–.050	.114	–.050	–.442	.660
	Preop. surgery	.051	.071	.076	.719	.474
	Med. history in general	–.155	.109	–.135	−1.420	.159
	Med. eye history	.044	.063	.072	.698	.487
	Documentation	.023	.098	.024	.235	.814
	Quality of laser coagulation	.091	.105	.089	.869	.387
	Amount of membrane peeling	.058	.054	.106	1.068	.288
	Consistency of surgeon	.088	.060	.167	1.452	.150
	Timing of cataract surgery	.164	.103	.177	1.598	.114
	P(D)VR stage	.228	.081	.328	2.812	.006
	Preop. fovea situation	.134	.059	.237	2.293	.024
	Amount of retino/retinectomies	.030	.069	.048	.437	.663
	Number and size of retinal hole area	–.094	.073	–.131	–1.287	.202
	Redetachment rate	.207	.150	.142	1.384	.170
	Axial length	.020	.068	.031	.300	.765

13	(Constant)	.952	.768	—	1.240	.218
	Consistency of surgeon	.081	.047	.155	1.717	.089
	P(D)VR stage	.218	.067	.313	3.270	.001
	Preop. fovea situation	.095	.052	.167	1.805	.074
	Redetachment rate	.277	.134	.190	2.071	.041

^a^Dependent variable: postoperative vision.

## Abbreviations

AC: Anterior chamber
AHFG: Anterior hyaloid fibrovascular proliferation
ERM: Epiretinal membrane
EVALP: Evaluation protocol
EVRS: European Vitreoretinal Society
Eyesi: Eye simulator (manufactured by VRmagic company)
f/u: Follow-up
g: Gauge
GRT: Great retinal tears
ILM: Inner limiting membrane
mPa·s: Millipascal seconds
med.: Medical
P(D)VR: Proliferative (diabetic) vitreoretinopathy
PFCL: Perfluorocarbon liquids
PPV: Pars plana vitrectomy
PVD: Posterior vitreous detachment
SLP: Scheimpflug lens photography
SO: Silicone oil
SOT: Silicone oil tamponade
SOP: Standard operation procedure
TRD: Tractional retinal detachment
VB: Vitreous body.
